# Learners to Leaders: Impact of Instructor Roles on Nursing Students’ Professional Development in Clinical Simulations

**DOI:** 10.3390/nursrep14040267

**Published:** 2024-11-25

**Authors:** Cristina García-Salido, Estel·la Ramírez-Baraldes, Daniel Garcia-Gutiérrez

**Affiliations:** 1 Departamento de Enfermería, Facultad de Ciencias de la Salud de Manresa, Universitat de Vic-Universitat Central de Catalunya (UVic-UCC), Av. Universitària, 4-6, 08242 Manresa, Spain; cgarcia@umanresa.cat (C.G.-S.); dgarcia04@umanresa.cat (D.G.-G.); 2 Grupo de Investigación en Simulación e Innovación Transformativa (GRIST), Universitat de Vic-Universitat Central de Catalunya (UVic-UCC), Av. Universitària, 4-6, 08242 Manresa, Spain; 3 Instituto de Investigación e Innovación en Ciencias de la Vida y de la Salud de la Cataluña Central (Iris-CC), 08500 Vic, Spain; 4 Unitat de Cures Intensives, Althaia Xarxa Assistencial Universitària de Manresa, 08243 Manresa, Spain

**Keywords:** clinical simulation, nursing students, instructor role, debriefing, non-technical skills, professional development, nursing education, qualitative study, feedback, Spain

## Abstract

Background/Objectives: Clinical simulation is a pivotal educational strategy in nursing, facilitating the integration of theoretical knowledge with practical skills in a safe environment. While the benefits of simulation in enhancing technical and non-technical competencies are well-documented, the transition of nursing students to the role of instructors within these simulations remains underexplored, particularly in the Spanish context. This study aims to investigate how assuming the instructor role in clinical simulations impacts the professional development of fourth-year nursing students. Methods: A qualitative study employing an interpretative phenomenological approach was conducted to deeply understand the experiences and perceptions of nursing students transitioning to instructors in clinical simulations. Nine fourth-year nursing students from the University of Vic—Central University of Catalonia (UVic-UCC) were purposively selected to ensure diverse perspectives. Data were collected through semi-structured interviews, a focus group, and non-participant observations, achieving data saturation. Thematic analysis, guided by Braun and Clarke’s methodology, was utilized to identify and organize emergent themes related to professional development and pedagogical competencies. Results: The transition to the instructor role significantly enhanced both technical and non-technical competencies among the participants. Key findings include improved leadership, decision-making, and effective communication skills. Student-led debriefing sessions were identified as crucial for fostering deeper reflection and enhancing the ability to provide and receive constructive feedback. Conclusions: Assuming the instructor role in clinical simulations offers substantial pedagogical benefits, enriching the professional development of nursing students by strengthening essential competencies. These findings underscore the value of integrating instructor roles within nursing education programs in Spain, suggesting that such practices can lead to more competent and confident healthcare professionals. This study was not registered.

## 1. Introduction

Nursing education varies considerably across the globe in terms of duration, structure, and pedagogical approaches, reflecting differences in cultural, economic, and healthcare policy frameworks. Nurses represent the largest group of healthcare professionals worldwide, comprising 59% of the global health workforce, and play a critical role in achieving universal health coverage (UHC) and the Sustainable Development Goals (SDGs) [1]. Despite their essential contributions, disparities in the quality and accessibility of nursing education exacerbate regional inequities in workforce distribution, hindering the provision of equitable and effective healthcare services [1].

In the United States, nursing programs are renowned for their advanced frameworks that integrate theoretical instruction, clinical practice, and competencies in leadership and healthcare management. These robust structures have established the United States as a global leader in nursing education, emphasizing leadership, research, and clinical expertise as core competencies [2]. Similarly, Canadian nursing education prioritizes a competency-based approach designed to cultivate clinical proficiency and leadership capabilities in response to the evolving demands of healthcare systems [3]. However, the increasing shortage of nursing faculty in high-income countries, including Canada, poses significant challenges to sustaining educational quality and necessitates targeted investments in academic infrastructure and workforce development [1,4].

In Europe, nursing education aligns with the principles of the Bologna Process, which standardizes academic frameworks to ensure comparability and compatibility of qualifications across countries. In Spain, the integration of nursing education into the Espacio Europeo de Educación Superior (EEES), enabled by Real Decreto 55/2005 [5], facilitated the transition from hospital-based training models to university-level curricula, fostering alignment with European academic standards. The Bologna framework has also supported the integration of simulation-based learning into nursing curricula across Europe, as evidenced by its implementation in Austria [6]. Austria’s competency-based, outcome-oriented nursing education ensures alignment with healthcare system demands, equipping graduates with critical leadership and decision-making skills. Nonetheless, variations in program duration and structure persist across Europe, reflecting diverse national healthcare policies and demands [7].

In Asia, countries such as Japan and South Korea demonstrate distinctive approaches to nursing education [2]. Japanese nursing programs combine clinical training with research-oriented curricula, fostering innovation in healthcare delivery. Meanwhile, South Korea integrates advanced technologies and interprofessional education into its four-year nursing programs, reflecting a commitment to collaborative practice and cutting-edge healthcare systems.

These global disparities underscore the need for harmonized strategies in nursing education to ensure the preparation of a highly skilled, adaptable nursing workforce. Within this context, clinical simulation has emerged as a transformative pedagogical approach that bridges gaps in theoretical knowledge and clinical practice, fostering the development of essential competencies in safe, controlled environments.

### 1.1. Clinical Simulation in Nursing Education

Clinical simulation is a transformative educational strategy that recreates healthcare scenarios in controlled environments, enabling students to practice, refine, and assess their skills without posing risks to real patients [8]. This methodology has become a cornerstone of modern healthcare education, bridging the gap between theoretical knowledge and clinical practice while promoting the development of essential technical and non-technical competencies [9,10].

Simulation allows students to confront complex clinical situations, fostering critical thinking, decision-making, effective communication, and teamwork skills [11,12,13]. These competencies are indispensable for ensuring safe, high-quality, and patient-centered care [14]. Additionally, simulation provides a secure environment for students to learn from mistakes, enhancing their confidence and preparedness for real-life clinical encounters [15]. High-fidelity simulation, in particular, has been identified as an effective method for developing practical clinical skills, offering immersive and realistic learning experiences [16].

Globally, simulation-based education has demonstrated its capacity to enhance student confidence and clinical performance across diverse contexts. In the United States and Canada, simulation has been extensively integrated into nursing curricula, effectively supplementing and even replacing traditional clinical hours without compromising learning outcomes [3,17]. Systematic reviews emphasize its effectiveness in improving both technical and soft skills, positioning simulation as a critical pedagogical tool in nursing education [9]. Longitudinal studies further highlight its reliability in maintaining educational quality even when traditional clinical placements are limited [3].

In Europe, the implementation of simulation varies significantly across countries, reflecting differences in curricula and healthcare demands. In Spain, simulation is gradually gaining prominence, despite challenges in standardization and access to resources [18]. Austria, aligned with the Bologna Process, has successfully integrated simulation into its competency-based nursing education, emphasizing measurable outcomes and skill acquisition [6]. These regional variations highlight both the opportunities and challenges associated with simulation adoption in diverse educational settings.

Beyond technical skill development, simulation plays a crucial role in reducing students’ anxiety while enhancing their confidence and self-efficacy. High-fidelity simulations, which closely mimic real-life clinical environments, have been particularly effective in achieving these outcomes [13,17]. Systematic reviews demonstrate how these simulations help students transition from academic settings to clinical environments with greater ease and confidence [15]. Furthermore, interprofessional simulation exercises foster collaboration across disciplines, preparing students for the complexities of modern healthcare teams [17].

### 1.2. Transition to Instructor Roles in Simulation-Based Education

A less explored but increasingly relevant dimension of simulation-based education is the transition of students to the role of instructors. This role shift allows students to engage in planning, directing, and debriefing simulations, fostering leadership, pedagogical skills, and reflective practice [19,20]. Evidence suggests that taking on instructional roles enhances students’ self-confidence and prepares them for future responsibilities as educators and mentors [20,21,22]. Student-led debriefing has been highlighted as a particularly effective approach to developing these competencies, as it encourages critical reflection and active engagement in peer learning [19,22].

Despite its growing adoption, the integration of simulation in nursing education is not without challenges. Variability in access to resources, differences in faculty training, and inconsistent implementation across institutions remain barriers to fully realizing its potential [9,18]. Faculty development programs are essential for equipping educators with the skills necessary to design and facilitate high-fidelity simulations effectively [22]. Additionally, standardized frameworks and guidelines, such as those proposed by the International Council of Nurses, are critical for ensuring consistency and quality in simulation-based education [14].

### 1.3. Study Context and Objectives

In the context of this study, clinical simulation serves as the foundation for innovative pedagogical strategies aimed at advancing nursing education. In the fourth year of the UVic-UCC nursing program [23], students can enroll in the elective course “*Advanced Clinical Simulation*”, representing a pivotal opportunity to assume instructional roles in simulation-based activities. Prior to this course, students participated as active participants in clinical scenarios and observers analyzing peer performance. This innovative course allows students to alternate between observer and instructor roles, fostering the development of leadership, pedagogical, and reflective skills essential for professional success.

Despite the increasing adoption of simulation in nursing education, there is a notable gap in the literature regarding how transitioning to instructor roles impacts the learning and professional development of nursing students, particularly in the Spanish and European contexts. Understanding this dynamic is crucial, as it could provide additional benefits in developing key competencies and improving the overall quality of nursing education. Understanding this dynamic is crucial, as it could provide additional benefits in developing key competencies and improving the overall quality of nursing education.

Therefore, the objective of this study is to explore the experiences and perceptions of fourth-year nursing students as they transition to instructor roles during simulation-based activities. Specifically, the study seeks to answer the following research questions:

▪ How do students perceive their transition to the role of instructor in clinical simulations?
▪ What technical and non-technical competencies are developed in this role?
▪ How does this experience influence their professional training and preparedness for clinical practice?

By addressing these questions, this research aims to contribute novel insights to the growing body of evidence on simulation-based education and inform the development of innovative strategies to enhance nursing education globally.

## 2. Materials and Methods

### 2.1. Study Design

A qualitative study with an interpretative phenomenological approach was conducted to deeply explore the perceptions and experiences of fourth-year nursing students regarding their participation in clinical simulations and their transition to the role of instructors. This approach is suitable for understanding how individuals interpret and assign meaning to their lived experiences within specific contexts [24]. The study aimed to capture the subjective and contextual dimensions of this phenomenon, allowing for a rich and detailed understanding of the changes experienced by the students when assuming the instructor role and its impact on their learning and professional development.

### 2.2. Elective Course: Advanced Clinical Simulation

The elective course “Advanced Clinical Simulation”, offered during the fourth year of the nursing program at the University of Vic—Central University of Catalonia (UVic-UCC), is a 3-credit course based on the European Credit Transfer and Accumulation System (ECTS), a standardized system within Espacio Europeo de Educación Superior (EEES) designed to facilitate the recognition and transfer of academic credits across Europe. This course is designed to immerse students in the fundamentals of simulation-based education. This course represents a pivotal transition in the students’ educational journey, as it allows them to alternate between the roles of observer and instructor during clinical simulation activities. The course integrates theoretical instruction on simulation pedagogy, scenario development, leadership, and feedback strategies with practical application, where students alternate between observer and instructor roles during simulation sessions, gaining a comprehensive understanding of simulation-based education.

As instructors, students take on a leadership role and are responsible for the following key tasks:

- Scenario planning: Creating realistic and clinically relevant cases that align with defined learning objectives.
- Simulation facilitation: Guiding peers through the simulation scenarios, ensuring the activity runs smoothly, addressing challenges in real time, and promoting active participation.
- Debriefing leadership: Leading structured, reflective discussions post-simulation, where they analyze participants’ performance, highlight areas for improvement, and provide constructive feedback to enhance learning outcomes.

### 2.3. Participants

The sample consisted of nine fourth-year nursing students, selected through purposeful sampling [25]. The inclusion criteria were as follows:

- Being enrolled in the fourth year of the nursing program.
- Having participated in clinical simulations as students since the first year.
- Having assumed the role of instructor in an elective course on clinical simulation during the fourth year.

Efforts were made to ensure heterogeneity in terms of gender and academic backgrounds to obtain a variety of perspectives. The sample size was determined based on the principle of data saturation, which was achieved when additional data collection no longer provided new insights relevant to the study objectives [26]. Saturation was reached after the ninth interview, as no new themes or codes were emerging, indicating that the sample size was sufficient to capture the diversity of experiences among participants.

### 2.4. Data Collection Procedures

Data were collected between March and May 2023, using three main techniques: individual semi-structured interviews, a focus group, and non-participant observation. These methods allowed for data triangulation, enhancing the validity and depth of the findings [27].

All data collection activities, including semi-structured interviews, the focus group, and non-participant observations, were conducted by a member of the research team with prior experience in qualitative research and training in data collection techniques. The researcher ensured methodological rigor throughout the process, aligning all activities with the study objectives and fostering a neutral and comfortable environment to encourage open and honest communication. The semi-structured interviews and focus group were guided by pre-designed thematic frameworks and open-ended questions to explore participants’ experiences in depth. Similarly, during the non-participant observations, the researcher acted as an external observer, maintaining a non-intrusive stance to objectively record interactions and behaviors. Structured observation sheets were used to document aspects such as scenario planning, facilitation strategies, and participant dynamics. These procedures followed established qualitative research standards, ensuring the credibility and depth of the data collected [25,26,27,28,29,30].

#### 2.4.1. Semi-Structured Interviews

Individual semi-structured interviews were conducted with each of the nine participants. An interview guide was developed based on the existing literature and the study objectives [20]. Open-ended questions allowed for an in-depth exploration of the students’ experiences and perceptions in their dual roles as students and instructors. The main thematic areas addressed included the following:

▪ Experiences and feelings during the transition from student to instructor role.
▪ Development of clinical and non-technical competencies through simulation.
▪ The role of debriefing and feedback in the learning process.
▪ Perceived challenges and benefits in participating in clinical simulations.

The interviews lasted between 40 and 60 min and were conducted in a private room within the faculty, ensuring a comfortable environment free from interruptions. All interviews were audio-recorded with the informed consent of the participants and transcribed verbatim for subsequent analysis.

#### 2.4.2. Focus Group

Following the individual interviews, a focus group was organized with the same nine participants to foster group discussion and exchange of experiences [29]. A discussion guide expanded on the topics addressed in the individual interviews, focusing on group dynamics and collective reflection on experiences in clinical simulation. The focus group lasted approximately 90 min and was conducted in an environment designed to promote trust and openness among participants. The session was recorded and fully transcribed.

#### 2.4.3. Non-Participant Observation

Non-participant observation was conducted during two sessions in which the students assumed the role of instructors in clinical simulations. The researcher positioned themselves as an external observer, without intervening in the activities, to objectively record the interactions and dynamics between instructors and students [30]. Structured observation sheets were used to record specific aspects:

▪ Planning and development of clinical cases by the instructors. Student instructors were responsible for both designing original simulation scenarios and organizing the logistical aspects of implementing these simulations. This involved creating realistic clinical situations, determining the objectives of each simulation, and ensuring that all necessary resources and materials were prepared in advance.
▪ Didactic strategies employed during the simulation and debriefing.
▪ Verbal and non-verbal interactions between instructors and students.
▪ Responses and behaviors of students to the feedback received.

The field notes obtained complemented the data from the interviews and focus group, providing an additional perspective and enriching the analysis.

### 2.5. Data Analysis

Data analysis was performed following Braun and Clarke’s thematic analysis approach [31], comprising six phases:

1. Familiarization with the Data: Multiple readings of the interview transcripts, focus group, and field notes were conducted to immerse in the content and capture significant nuances.
2. Generating Initial Codes: Relevant elements in the data were identified and coded using the Atlas.ti software, version 24 (Lumivero Company, Berlin, Germany), specialized for qualitative data analysis. Twenty-five initial codes were generated, representing emerging ideas and patterns. Some of the most significant codes included:
  - “Application of theoretical knowledge”
  - “Confidence in clinical skills”
  - “Leadership development”
  - “Constructive feedback”
  - “Anxiety during simulations”
  - “Responsibility as an instructor”
  - “Inadequate preparation”
  - “Impact of debriefing”
  - “Peer motivation”
  These codes represented themes that repeatedly emerged throughout the interviews and observations, allowing for the organization of students’ perceptions regarding their experiences in clinical simulations, both as students and instructors.
3. Searching for Themes: The codes were grouped into seven main categories that encompassed the most salient aspects of the study:
  3.1. Simulation as a Bridge between Theory and Practice: Includes codes related to the application of theoretical knowledge in a safe environment.
  3.2. Transition to the Instructor Role: Groups codes related to perceived changes when moving from student to instructor.
  3.3. Confidence and Clinical Competence: Covers aspects of self-perception regarding students’ technical and clinical skills.
  3.4. Debriefing and Feedback: Includes codes related to the value of post-simulation feedback and guided debriefing.
  3.5. Challenges in Motivation and Preparation: Addresses issues related to lack of commitment or preparation by students.
  3.6. Development of Non-Technical Skills: Groups codes reflecting the impact on transversal competencies such as leadership, communication, and decision-making.
  3.7. Emotional Impact and Stress: Related to the emotional impact experienced by students in simulations under observation.
4. Reviewing Themes: The identified categories were reviewed and refined to ensure that they accurately represented the data and that there was coherence within and between themes. Any overlapping or redundant categories were adjusted.
5. Defining and Naming Themes: The seven categories were organized into three broad metacategories that summarized the main research axes:
  - Development of Technical and Non-Technical Competencies through Simulation: Encompasses the categories “Simulation as a Bridge between Theory and Practice”, “Confidence and Clinical Competence”, and “Development of Non-Technical Skills”.
  - The Pedagogical Impact of Transitioning to the Instructor Role: Includes the categories “Transition to the Instructor Role” and “Debriefing and Feedback”, highlighting how the experience of being an instructor reinforces students’ competencies and enhances their critical analysis skills.
  - Emotional and Organizational Challenges in Simulation: Covers “Challenges in Motivation and Preparation” and “Emotional Impact and Stress”, addressing the difficulties faced by students in both planning and executing simulations.
  This systematic process of coding, categorization, and grouping into metacategories allowed for a coherent organization of information, ensuring that qualitative data were analyzed rigorously and systematically.
6. Producing the Report: The findings were integrated into a cohesive narrative, utilizing verbatim quotes from participants and field notes to support the themes and ensure that students’ experiences and perceptions were solidly integrated into the final analysis. The thematic analysis was iterative, allowing for constant revision of the identified themes based on the richness and coherence of the data.
  To ensure the trustworthiness of the analysis, three strategies were employed [24]:
  ▪ Data Triangulation: Findings from the interviews, focus group, and observations were compared and contrasted to identify consistencies and discrepancies [32]. This triangulation enhances the credibility of the results by cross-verifying information from multiple sources.
  ▪ Peer Review: An independent researcher reviewed the coding process and emerging categories, providing an additional perspective and ensuring dependability. This collaborative approach helps to minimize potential biases in data interpretation.
  ▪ Member Checking: Preliminary findings were shared with some participants to confirm the interpretation of their experiences and ensure that they accurately reflected their perceptions [33]. This process strengthens the confirmability of the study by involving participants in validating the results.

### 2.6. Ethical Considerations

The study was approved by the UVic-UCC Research Ethics Committee, which issued a favorable report for the research project titled “Transferability of the knowledge acquired with simulation methodology to clinical practices in the nursing degree” (code: 141/2021). Ethical principles established in the Declaration of Helsinki and guidelines for research involving human subjects were followed [34]. Participation in this study was entirely voluntary and free, with no implications for the participants’ grades in the course. Prior to data collection, participants were provided with detailed information about the study objectives, data collection procedures, and their right to withdraw at any time without repercussions. All participants signed an informed consent form. Confidentiality and anonymity were ensured by using pseudonyms in transcriptions and analyses. The collected data were securely stored, and only the research team had access to them. Non-participant observation was conducted without interfering in the students’ activities, respecting their autonomy and privacy.

## 3. Results

The qualitative analysis of the data obtained through semi-structured interviews, a focus group, and non-participant observations identified seven main categories, which were grouped into three metacategories: (1) development of technical and non-technical competencies, (2) pedagogical impact of the transition to the instructor role, and (3) challenges and improvement proposals in clinical simulation. Below, we present detailed findings for each category, integrating the importance of debriefing and constructive feedback within the corresponding metacategory, supported by participants’ verbatim quotes [24].

### 3.1. Development of Technical and Non-Technical Competencies

#### 3.1.1. Clinical Simulation as a Bridge Between Theory and Practice

All participants concurred that clinical simulation is essential for bridging theoretical knowledge with real-world clinical practice. Simulation was perceived as a tool that facilitates the application of learned concepts in a safe and controlled environment, allowing them to consolidate clinical skills before facing real-life situations. For instance, one student stated: “*Simulation is a bridge that connects knowing… with doing*”.

This metaphor reflects the perception of simulation as a crucial intermediary between theory and practice. Another student reinforced this idea by highlighting the value of learning through practice: “*I think it’s a very good way to learn through practice*”.

Simulation was also recognized for its impact on the retention and application of specific procedures. A student working as a nursing assistant mentioned: “*The day I have to perform CPR*, *of course*, *I did it in simulations and reviewed it. It refreshes your knowledge*”.

Moreover, simulation increased students’ confidence in facing real clinical situations. Another student highlighted how a simulation in the Intensive Care Unit (ICU) prepared her for her practicum: “*The whole process has been quite a good learning experience*”.

Similarly, one student reflected on the usefulness of simulation in strengthening her skills: “*It’s a course that was super useful and super important for me to carry out the other practicums later on*”.

In the focus group, the relevance of simulation in specific clinical situations was emphasized: “*I encountered it in clinical practice*, *exactly as you taught it in the simulation*”.

#### 3.1.2. Development of Non-Technical Skills: Leadership, Communication, and Decision-Making

Simulation significantly contributed to the development of essential non-technical skills for clinical practice. Participants reported improvements in leadership, effective communication, and decision-making under pressure. One student remarked: “*Being an instructor gives you a different perspective because you have to guide*, *provide feedback*, *and control the process*”.

This role change allowed students to understand the importance of coordinating and leading a team, fundamental skills in multidisciplinary clinical settings. Another student highlighted: “*It helps you see what your role would be in a group of eight people*, *how you can lead or where you need to help*”.

The ability to anticipate problems and complications was also mentioned as a skill developed when acting as an instructor: “*As an instructor*, *you have to see not only what’s happening but what could happen next*, *anticipate complications*”.

### 3.2. Pedagogical Impact of the Transition to the Instructor Role

#### 3.2.1. Deep Understanding of Simulation Planning and Execution

Students expressed that assuming the instructor role allowed them to appreciate the complexity and effort involved in the preparation and execution of clinical simulations: “*I didn’t know that to create a clinical case*, *you had to consider so many aspects*”.

Prior planning was identified as a critical component for the success of simulations. One student emphasized: “*Seventy or eighty percent of the simulation is preparing it beforehand*”.

This realization underscores the importance of thorough preparation to ensure an effective learning experience.

#### 3.2.2. Development of Pedagogical Competencies and Critical Reflection

The instructor role facilitated the development of pedagogical skills, especially concerning conducting debriefings and providing constructive feedback. Students recognized that leading the debriefing was a significant responsibility requiring communicative abilities and critical analysis: “*For me*, *the most challenging part is the debriefing… because depending on how you say it*, *the person may not take it the best way*”.

Debriefing emerged as an essential component of the simulation process, valued as the moment when the most significant learning occurs through reflection and critical analysis: “*Debriefing is when… you realize what we did wrong or right*, *and you can learn from it*”.

Students acknowledged the challenge of providing effective and sensitive feedback, which is essential for promoting meaningful learning among peers. One student described debriefing as a fundamental and spontaneous phase: “*Debriefing… is super improvised at the moment with what happened just a minute ago*”.

The manner in which feedback was delivered influenced students’ confidence and willingness to improve: “*When the feedback is positive*, *even if you’ve made mistakes*, *you feel more confident to try again*”.

The use of additional resources, such as reviewing videos of the simulations, was suggested to enhance self-analysis and reflection: “*Watching the simulation video helped me see what I could improve; it’s like a deeper self-analysis*”.

### 3.3. Challenges and Improvement Proposals in Clinical Simulation

#### 3.3.1. Student Motivation and Engagement

A recurrent challenge identified was the lack of motivation and engagement from some students during simulations, negatively impacting the quality of learning: “*We students should take it more seriously to get more out of that knowledge*”.

Generating curiosity and interest in clinical cases was suggested as a strategy to enhance participation: “*I think the key would be to generate curiosity… because that way we participate more and are more attentive*”.

In the focus group, it was highlighted that prior preparation and interest in the topics increased student engagement: “*If the topic interests you*, *you’ll be more attentive and internalize it better*”.

#### 3.3.2. Preparation as a Key Factor for Simulation Success

Prior preparation was identified as a determining factor in the effectiveness of simulations and in students’ self-directed learning: “*The success of the simulation depends on how well you’ve prepared beforehand*”.

Some students admitted difficulties in maintaining consistent preparation, affecting the development of simulations: “*I’m not a very consistent person in that sense… I’ve rarely prepared*”.

#### 3.3.3. Improvement Proposals: Early Integration and Increased Frequency of Simulations

Participants suggested that clinical simulation should be integrated from the early years of the curriculum and conducted more frequently to maximize its educational impact. While the curriculum at UVic-UCC includes clinical simulations from the first year of the nursing program, some students expressed a desire for even earlier or more extensive exposure to these activities. This feedback likely reflects a perception of the value of simulations as an essential educational tool. Students emphasized the importance of being exposed to simulation-based learning at the very start of their studies, along with a higher frequency of simulations throughout the program.

One participant reflected on this by stating: “*I would include it in the first year… explain what it’s about*, *how it’s done*, *and then put it into practice. Maybe it would change a bit how we see it*”.

Another student highlighted the importance of repeated exposure to simulation-based scenarios to consolidate learning: “*Repeating simulations*, *especially in complex cases*, *would be very useful to better integrate knowledge*”.

#### 3.3.4. Application of Simulation in the Workplace

Finally, students proposed implementing clinical simulations in the workplace to improve coordination and teamwork among professionals from different disciplines: “*It would be interesting to do periodic simulations in hospitals*, *like a monthly drill*”.

Interprofessional simulation was identified as a tool to enhance communication and response in critical situations: “*Doing simulations with various professionals is key to improving coordination*, *especially in critical situations*”.

## 4. Discussion

This qualitative study explored in depth the transition of fourth-year nursing students to the role of instructors in clinical simulations, an area scarcely investigated in nursing education. The findings reveal that assuming this role has a significant impact on the development of both technical and non-technical competencies, enriching the professional formation of the students. The results are analyzed in relation to the existing literature, implications for practice are discussed, and the study’s limitations are outlined.

### 4.1. Clinical Simulation as a Bridge Between Theory and Practice

The results confirm that clinical simulation is an effective tool for connecting theoretical knowledge with real clinical practice, allowing students to apply and consolidate their competencies in a safe and controlled environment. This finding is consistent with previous research highlighting the value of simulation in nursing education [35]. Simulation facilitates the development of clinical skills, enhances critical reasoning, and increases students’ confidence when facing real-life situations [36,37].

However, this study offers a new perspective by demonstrating that when students assume the role of instructors, the effectiveness of simulation is amplified. The preparation and execution of simulations by student instructors deepen their understanding of content, promote more active learning, and reinforce the transfer of knowledge to clinical practice [38,39]. This finding suggests that involving students in teaching roles within simulation can enhance educational benefits and should be considered in curriculum design.

### 4.2. Impact of Transitioning to the Instructor Role

The transition to the instructor role emerged as a key factor in developing pedagogical competencies and non-technical skills such as leadership, effective communication, and decision-making. Student instructors developed a deeper understanding of clinical and educational processes, improving their ability to lead teams and provide constructive feedback. These results align with studies demonstrating that active participation in teaching enhances students’ interpersonal skills and self-confidence [39,40].

Moreover, the instructor role fostered greater critical reflection and self-awareness. Students had to analyze both their peers’ performance and their own, promoting the development of metacognitive skills essential for professional practice [41,42]. This level of reflection is fundamental in forming professionals capable of continuously evaluating and improving their clinical practice.

### 4.3. Debriefing and Constructive Feedback as Key Components of Learning

Debriefing was identified as an essential element in the simulation process, especially when students assumed the role of facilitators. Leading debriefing sessions allowed student instructors to develop skills in communication, critical analysis, and feedback provision—competencies crucial in clinical settings [43,44]. This finding reinforces evidence that effective debriefing enhances learning, fosters reflective thinking, and contributes to the development of non-technical skills [45,46].

This study highlights that student instructors faced challenges when providing feedback, which allowed them to learn how to communicate observations constructively and sensitively. This experience is valuable for their future professional practice, where effective communication and the ability to provide and receive feedback are essential for quality improvement and patient safety [47,48].

The pivotal role of debriefing in simulation-based learning was evident in our findings, particularly when students assumed facilitator roles. Cleaver et al. (2021) demonstrated that structured debriefing enhances clinical judgment and competence among nursing students [49]. Similarly, our participants reported that leading debriefing sessions fostered critical analysis and reflective thinking, essential for professional practice. This suggests that incorporating structured debriefing frameworks could further amplify the educational benefits of simulations.

### 4.4. Challenges in Motivation and Preparation

Motivation is a critical determinant of learning outcomes in simulation-based education. Our study identified challenges related to student motivation and preparation, which affected the effectiveness of simulations and collective learning. This aligns with Cheng et al. (2021), who emphasize that understanding and addressing motivational factors are essential for enhancing student engagement and maximizing the benefits of simulation activities [50]. Implementing strategies that consider intrinsic and extrinsic motivators could therefore enhance participation and learning efficacy.

Despite the observed benefits, challenges related to student motivation and preparation were identified. The lack of commitment and preparation among some participants affected the effectiveness of simulations and collective learning. This issue has been reported in other studies, where inadequate prior preparation limits active participation and reduces the educational benefits of simulation [51,52].

Students suggested strategies to improve motivation, such as early integration of simulation into the curriculum and increasing the frequency of sessions. They also emphasized the need to generate curiosity and interest in clinical cases to encourage more active participation. These proposals are consistent with the literature highlighting the importance of curriculum design that promotes student engagement and responsibility in their own learning [53,54,55,56].

### 4.5. Implications for Educational Practice

The findings of this study have significant implications for nursing education. Integrating opportunities for students to assume instructor roles in clinical simulations can enrich their professional development by fostering critical competencies essential for practice. Educational institutions should consider incorporating instructor roles for advanced students, allowing senior students to design and facilitate simulations. This approach not only enhances their learning experience but also cultivates leadership and pedagogical skills. Additionally, promoting preparation and active participation is crucial; implementing strategies that encourage prior preparation and student commitment—such as specific assignments and formative assessments—can lead to more effective learning outcomes. Furthermore, integrating simulation progressively throughout the curriculum is recommended. Introducing simulation activities from the early years and gradually increasing their complexity can significantly improve the acquisition of both clinical and non-technical competencies. Lastly, advancing effective debriefing practices is essential. Training both students and instructors in debriefing techniques can enhance the quality of reflections and resultant learning, ensuring that the simulations provide maximum educational benefit.

### 4.6. Limitations of the Study

This study has several limitations. The sample was limited to nine students from a single institution, which may affect the generalizability of the results. Additionally, the qualitative approach is based on participants’ subjective perceptions, and while it provides deep understanding, it does not allow quantitative measurement of the impact of transitioning to the instructor role.

The potential influence of the researcher during data collection and analysis must also be considered, despite measures taken to minimize bias, such as method triangulation and the use of verbatim quotes. Future research could incorporate larger and more diverse samples and employ mixed methodologies to validate and expand these findings.

### 4.7. Recommendations for Future Research

Future studies should explore the following:

- The long-term impact of the instructor experience on graduates’ clinical performance and their integration into the workplace.
- Effective strategies to improve student motivation and preparation in clinical simulations.
- The application of interprofessional simulations, involving students from different health disciplines to enhance collaboration and communication in multidisciplinary teams.
- Quantitative analysis of the impact of transitioning to the instructor role on indicators such as academic performance, competency acquisition, and student satisfaction.

## 5. Conclusions

This qualitative study has demonstrated that transitioning nursing students to the instructor role within clinical simulations has a significant impact on their professional development. By assuming this role, students enrich their education by strengthening both technical and non-technical competencies, including skills in leadership, effective communication, and critical reflection.

The experience of acting as instructors allows students to deepen their understanding of clinical and educational processes, fostering greater self-confidence and preparedness to confront the challenges of real-world clinical practice. Moreover, student-led debriefing emerges as a pivotal component that facilitates the consolidation of learning and the development of essential competencies for professional practice.

These findings could have an impact on nursing education by informing curriculum development and teaching strategies. This practice can contribute to forming more competent and confident professionals, capable of responding effectively to the complex demands of the contemporary healthcare environment.

It is imperative that educational institutions consider these results in the design and enhancement of their curricula, promoting methodologies that foster active learning, student engagement, and the comprehensive development of competencies necessary for high-quality healthcare delivery.

## Data Availability

The data presented in this study are available on reasonable request from the corresponding author. Due to privacy and ethical restrictions, the data are not publicly accessible. Access will be provided in a manner that ensures the confidentiality of the participants and complies with ethical guidelines.

## Public Involvement Statement

No public involvement in any aspect of this research.

## Guidelines and Standards Statement

This manuscript was drafted against the COREQ (Consolidated Criteria for Reporting Qualitative Research) guidelines for qualitative research.
